# Protective effects of Gypenoside XVII against cerebral ischemia/reperfusion injury via SIRT1-FOXO3A- and Hif1a-BNIP3-mediated mitochondrial autophagy

**DOI:** 10.1186/s12967-022-03830-9

**Published:** 2022-12-27

**Authors:** Weijie Xie, Ting Zhu, Shuxia Zhang, Xiaobo Sun

**Affiliations:** 1 Research Center for Pharmacology and Toxicology, Institute of Medicinal Plant Development, Peking Union Medical College, Chinese Academy of Medical Sciences, Beijing, 100193 China; 2 Key Laboratory of Material Basis and Resource Utilization of Chinese Herbal Medicine, Beijing, 100193 China; 3 State Administration of Traditional Chinese Medicine Key Laboratory of Efficacy evaluation of Traditional Chinese Medicine in intervention of disorders of glucose and Lipid Metabolism, Beijing, 100193 China; 4 Institute of Neuroregeneration & Neurorehabilitation, Department of Pathophysiology, School of Basic Medicine, Qingdao University, Qingdao, 266071 China; 5 Shanghai Mental Health Centre, School of Medicine, Shanghai Jiao Tong University, Shanghai, 200011 China

**Keywords:** Gypenoside XVII, Cerebral ischemia/reperfusion injury, FOXO3A, BNIP3, Mitochondrial autophagy

## Abstract

**Background:**

Mitochondrial autophagy maintains mitochondrial function and cellular homeostasis and plays a critical role in the pathological process of cerebral ischemia/reperfusion injury (CIRI). Whether Gypenoside XVII (GP17) has regulatory effects on mitochondrial autophagy against CIRI remains unclear. The purpose of this study was to investigate the pharmacodynamic effects and mechanisms of GP17 on mitochondrial autophagy after CIRI.

**Methods:**

A rat middle cerebral artery occlusion/reperfusion (MCAO/R) model was used to assess the effects of GP17 against CIRI and to explore the underlying mechanisms. An oxygen-glucose deprivation/reoxygenation (OGD/R) cell model was used to verify the ameliorative effects on mitochondrial damage and to probe the autophagy pathways involved in combating neural injuries.

**Results:**

The in vivo results showed that GP17 significantly improved mitochondrial metabolic functions and suppressed cerebral ischemic injury, possibly via the autophagy pathway. Further research revealed that GP17 maintains moderate activation of autophagy under ischemic and OGD conditions, producing neuroprotective effects against CIRI, and that the regulation of mitochondrial autophagy is associated with crosstalk between the SIRT1-FOXO3A and Hif1a-BNIP3 signalling pathway that is partially eliminated by the specific inhibitors AGK-7 and 2-ME.

**Conclusion:**

Overall, this work offers new insights into the mechanisms by which GP17 protects against CIRI and highlights the potential of therapy with Notoginseng leaf triterpene compounds as a novel clinical strategy in humans.

**Supplementary Information:**

The online version contains supplementary material available at 10.1186/s12967-022-03830-9.

## Introduction

Stroke, especially ischemic stroke, is one of the leading causes of death worldwide. Stroke is mainly caused by cerebral ischemia/reperfusion injury (CIRI). In the early stage of ischemia, ischemia causes insufficient NAD^+^ levels and a decrease in the ratio of NAD^+^/NADH, which directly impairs H^+^ transmission in the oxidative respiratory chain and results in insufficient intracellular ATP synthesis; these effects lead to mitochondrial damage and energy metabolism disorders [1–3]. Nicotinamide phosphoribosyltransferase (NAMPT) is a rate-limiting enzyme in the NAD synthesis pathway in humans [4]. NAMPT can increase ischemic tolerance and improve mitochondrial energy metabolism during ischemia, suggesting that NAMPT may be a key target for the prevention and treatment of ischemic stroke. Searching for natural active substances and compounds that can effectively inhibit mitochondrial damage and alleviate neuronal apoptosis and necrosis, as well as exploring the mechanisms of these substances with respect to NAMPT targets, are popular research topics related to the prevention and treatment of ischemic stroke.

Autophagy is a conserved process of cellular self-digestion and catabolism in which lysosomes are responsible for degrading damaged proteins and organelles to maintain cellular homeostasis and normal function [5, 6]. As a homeostasis regulation mechanism in vivo, autophagy has been shown to participate in the entire process of ischemic stroke through pathways such as the PKC/JNK, PI3K/AKT-mTOR, and AMPK/mTORC1 pathways [6, 7]. Under ischemic and hypoxic conditions, autophagy is important for maintaining intracellular environmental stability and self-renewal ability, as it removes damaged organelles and abnormal proteins in cells. Thus, regulation of autophagy is regarded as a key strategy against CIRI.

BNIP3 is an extramitochondrial membrane protein that is expressed at low levels under normal physiological conditions. In the hypoxic environment, activated Hif-1α upregulates the expression of BNIP3, causing Beclin1 release. The enhancement of Beclin-1 expression can be used as an important indicator to evaluate the increase of autophagy level [8]. Thus, Hif-1α/BNIP3 pathway may be a novel target in the prevention and treatment for ischemic injury.

**Fig. 1:**
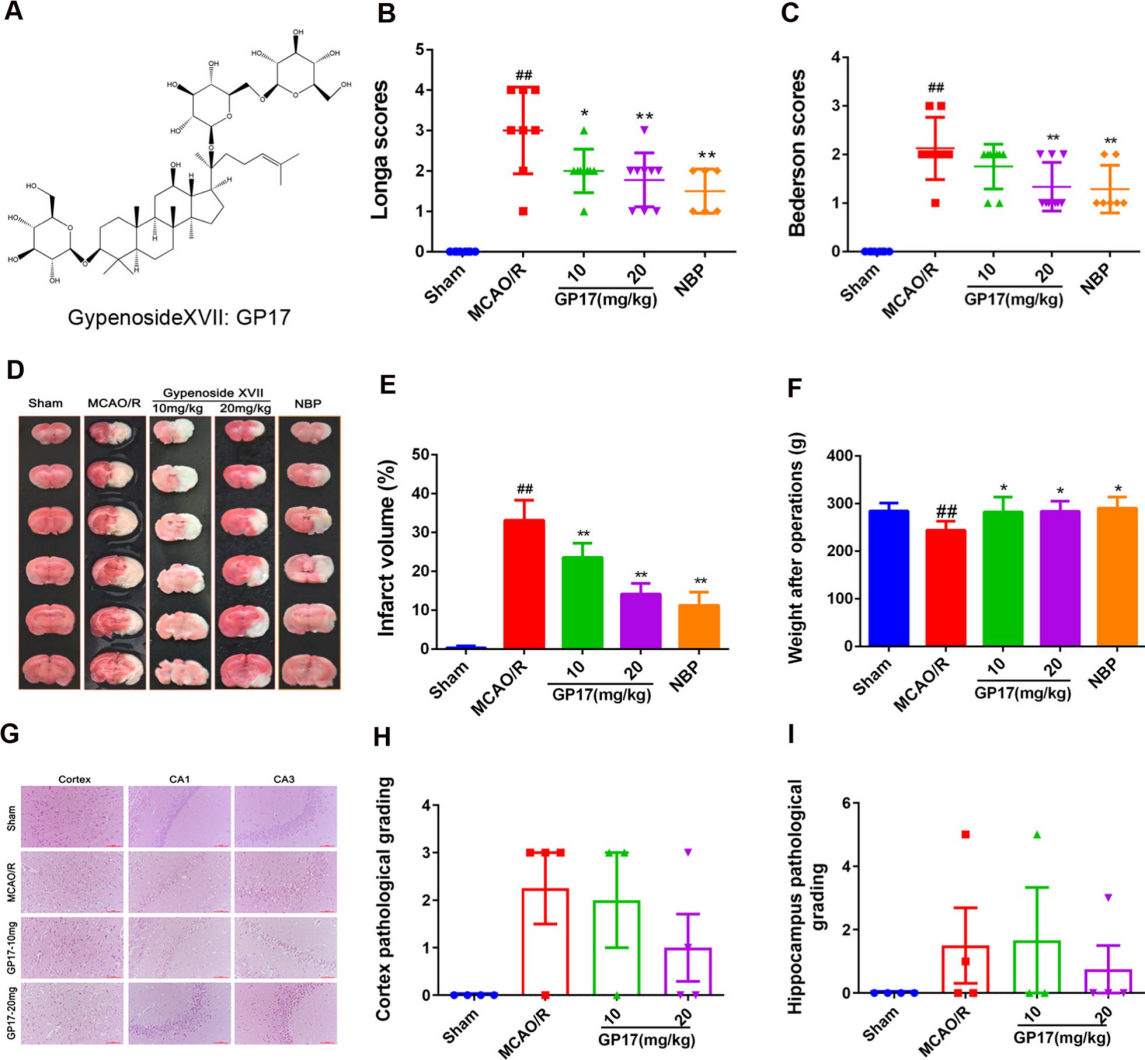
Effects of GP17 on infarct volume, neurological deficit scores and neuronal pathological changes in MCAO/R injury rats. **A** Chemical structure of GP-17. **B** and **C** Neurological deficit scores in all groups (n=10). **D** Representative images of TTC-stained brain sections from the Sham-operated or GP17-treated animals collected 24 h after infarction; Red tissue is healthy; white tissue is infarcted. **E** Quantitative analysis of the infarct volume (n=3–5). **F** Rat weight in all groups (n=6–10). **G** Representative images (X400) of H/E staining performed in cortex and hippocampus CA1, CA3 regions from ischemic brains, scale bar =100 μm. H and I, Quantitative analysis of pathological grading scores (n=3–4). Statistical comparisons were performed as follows: one-way ANOVA with Dunnett’s multiple comparisons test for (**B**, **C**, **E** and **F**); Wilcoxon test for (**H** and **I**); ^*^P<0.05, ^**^P<0.01 versus MCAO/R group; ^#^P<0.05, ^##^P<0.01 versus Sham group

Due to the complexity of the pathogenesis of ischemic stroke, the limitations of therapeutic drugs and the very large international and domestic needs, an increasing number of researchers are studying traditional Chinese medicines as important ways to treat ischemic stroke [9–11]. Notoginseng leaf triterpenes (PNGL), a total saponin extract of *Panax notoginseng* stems and leaves, attenuates neuronal apoptosis [12] and ameliorates mitochondrial oxidative injury caused by ischemia [13, 14]. Gypenoside XVII (GP17; the chemical structure is shown in Fig. 1A), a unique component of PNGL, exhibits many pharmacological effects through suppression of endothelial apoptosis, oxidative stress [15] and endoplasmic reticulum stress-induced mitochondrial injury [16]. Our previous studies have demonstrated that GP17 attenuates Aβ25–35-induced parallel autophagic and apoptotic cell death [17]. Moreover, GP17 can enhance lysosome biogenesis and accelerate autophagic clearance of amyloid-β through TFEB activation [18]. But its neuroprotective role in regulating autophagy has not been investigated in the context of ischemic stroke, and it is unclear whether GP17 and active saponin of Panax Notoginseng stems regulate autophagy via Hif-1α/BNIP3 pathway.

The mechanisms by which GP17 regulates mitochondrial protection in the NAMPT-mediated pathway have been elucidated[13, 14]. The present study further focused on the downstream pathways. Based on the previous searches and reports, it was hypothesized that the significant protective effects of GP17 against ischemic stroke might be exerted via autophagy via Hif-1α/BNIP3 pathway or NAMPT-mediated pathway, namely SIRT1/2/3. Thus, in this work, we further evaluated the effects of GP17 on CIRI in rats subjected to middle cerebral artery occlusion/reperfusion (MCAO/R) and SH-SY5Y cells subjected to oxygen-glucose deprivation/reoxygenation (OGD/R), and explore the underlying mechanisms. The purpose of this study was to illuminate the regulatory effects and the underlying molecular pathway of GP17 on mitochondrial damage and autophagy after ischemic stroke.

## Results

### GP17 reduces cerebral infarction, improves neurological deficit scores and alleviates neuronal pathological damage in rats after ischemia

In the therapeutic efficacy study on MCAO/R rats, GP17 pretreatment at 10 and 20 mg/kg notably decreased the Longa scores and Bederson scores 24 h after ischemia (Fig. 1B, C). In agreement with these findings, administration of intraperitoneal (i.p.) GP17 at 10 and 20 mg/kg 7 days before ischemia significantly reduced the infarct volume (Fig. 1D, E). GP17 (20 mg/kg)-treated rats exhibited neurological deficit scores similar to those of dl-3-n-butylphthalide (NBP)-treated rats. Accordingly, GP17-treated rats displayed better recovery of body weight than MCAO/R rats (Fig. 1F). At a dose of 20 mg/kg, GP17 also reduced the occurrence of pyknotic nuclei in the cortex (Fig. 1G, H) and hippocampal (Fig. 1G, I) regions.

In addition, GP17 (20 mg/kg) pretreatment significantly reduced the expression levels of the inflammatory cytokines IL-6, TNF-α, and MCP-1 and the oxidative cytokines T-AOC and 4-HNE (Additional file 1: Figure S1A–E).

### GP17 protects against CIRI by regulating mitochondrial autophagy in vivo

**Fig. 2:**
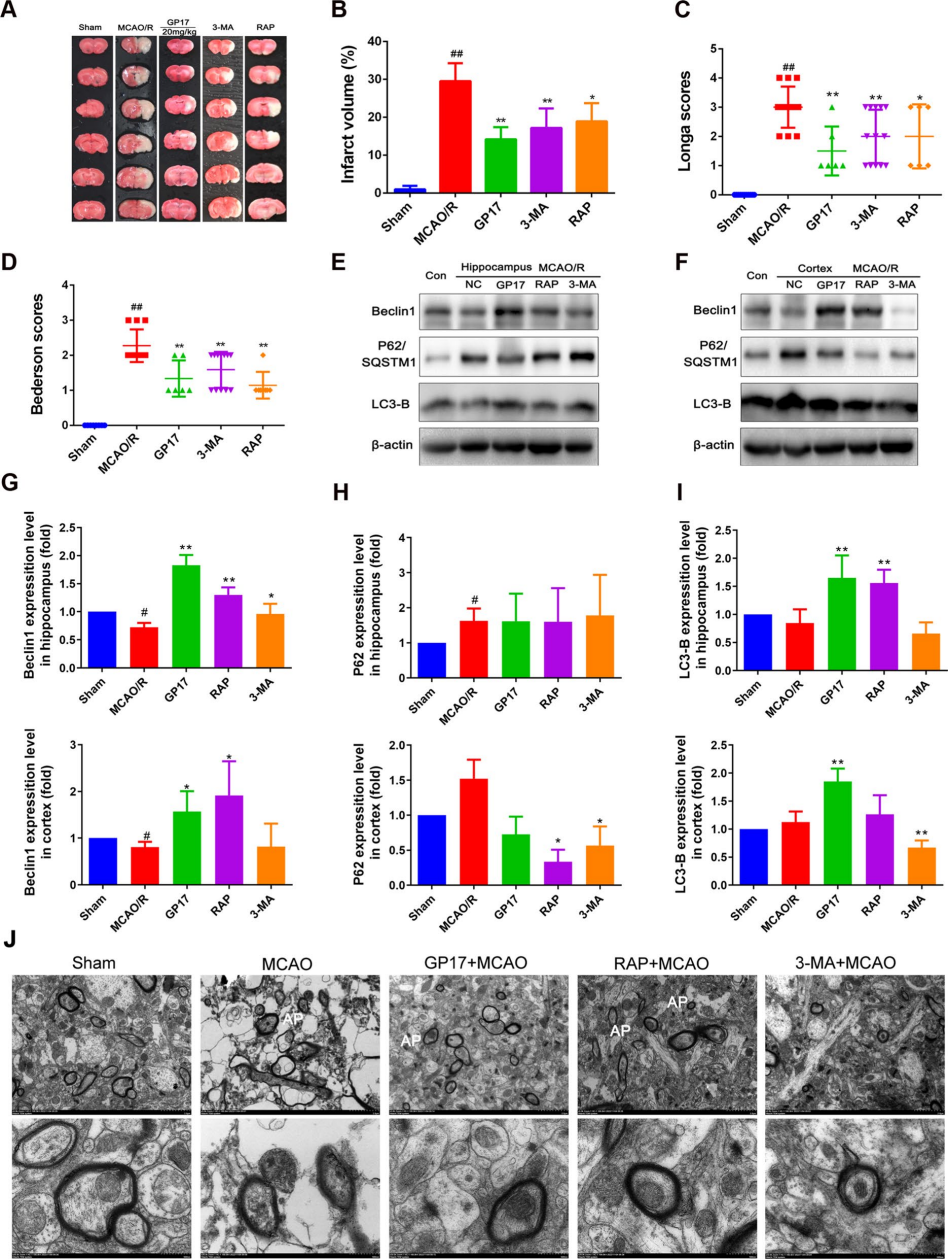
Effects of GP17, the autophagy activator RAP and the autophagy inhibitor 3-MA on infarct volume, neurological deficit scores, accumulation of autophagic vacuoles, and the Beclin1-LC3-P62 signalling pathway in rats with MCAO/R injury. **A** Representative images of TTC-stained brain sections from sham-operated or GP17-treated animals collected 24 h after infarction. Red tissue is healthy; white tissue is infarcted (n=3–6). **B** Quantitative analysis of the infarct volume (n=3). **C** and **D**. Neurological deficit scores in all groups (n=6–12). **E** and **F** The protein bands of LC3-B, Beclin1, and P62 in the ischemic brain sections were examined by western blot analysis. **G**–**I**. The relative expression levels of LC3, Beclin1, and P62 were quantified and analysed by using Gel-Pro analyser software (n=3–6). J. Accumulation of autophagic vacuoles (AP) was examined using a TEM; 5000, scale bars=2 μm, 20,000, scale bars=500 nm. Statistical comparisons were performed as follows: one-way ANOVA with Dunnett’s multiple comparisons test for (**B**, **C**, **D** and **I**); unpaired t test for (**G** and **H**); ^*^P<0.05, ^**^P<0.01 versus MCAO/R group; ^#^P<0.05, ^##^P<0.01 versus Sham group

To investigate the regulatory effect on mitochondrial autophagy in an acute model of ischemia, rapamycin (RAP), an autophagy inducer, and 3-MA, an autophagy inhibitor, were administered intraperitoneally 7 days before ischemia. Interestingly, both RAP (200 µM) and 3-MA (2 mM) significantly reduced the infarct volume (Fig. 2A, B) and neurological deficit scores (Fig. 2C, D). Furthermore, the effects of GP17 on the infarct volume and neurological deficit scores were stronger than those of 3-MA and RAP.

To further assess whether the protective mechanism of GP17 is related to regulation of mitochondrial autophagy, we used western blotting to analyse the expression changes in autophagy signalling pathway-related proteins. Our western blot results confirmed that GP17, RAP and/or 3-MA increased Beclin1 and LC3-B expression and inhibited P62 expression in hippocampal and/or cortical tissues (Fig. 2E–I). In addition, we processed cerebral cortex tissue of rats treated with GP-17, RAP, and 3-MA by transmission electron microscopy (TEM). TEM revealed that MCAO/R induced the accumulation of autophagosomes, whereas GP-17, RAP and 3-MA treatment enhanced the formation of several autophagic vacuoles (Fig. 2J). These data suggest that inhibiting excessive levels of autophagy and maintaining an appropriate level of autophagy can help improve brain injury and that GP17 may play a partial protective role through autophagy.

In addition, compared with that in the OGD/R + GP17 model group, the apoptosis level in the 3-MA (inhibitor) group was decreased, indicating that autophagy activation in the acute phase of ischemia and hypoxia can attenuate the apoptosis caused by ischemia. Furthermore, the RAP (inducer) group showed greater inhibition of neuronal cell apoptosis than the OGD/R group (Additional file 1: Figure S1F), suggesting that GP17 may exert a protective effect on ischemic neuronal cells in part through mitochondrial autophagy regulation.

### **GP17 regulates mitochondrial autophagy in rats after ischemia through activation of the Hif1α-BNIP3 and SIRT1/2-FOXO3A signalling pathways**

**Fig. 3:**
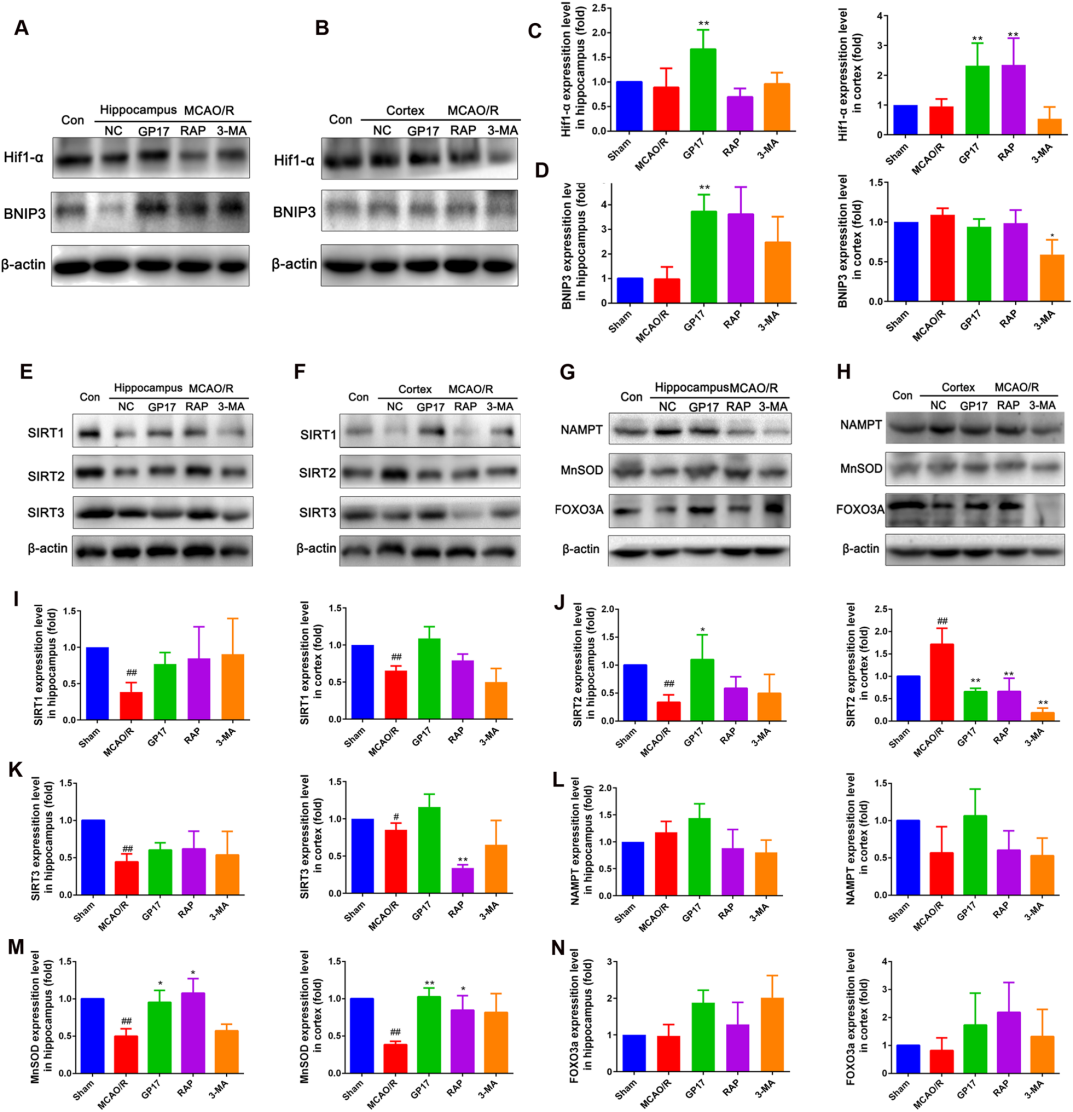
Effects of GP17 on the Hif1α-BNIP3 and SIRT1/2-FOXO3A signalling pathways and thus on the mitochondrial autophagy signalling pathway in rats with MCAO/R injury. **A** and **B** The protein bands of HIF1α-BNIP3 in ischemic brain sections were examined by western blot analysis. **C** and **D** The relative expression levels of Hif1α and BNIP3, respectively, were quantified and analysed by using Gel-Pro analyser software. **E** and **F** The protein bands of SIRT1/2/3 in the ischemic brain sections were examined by western blot analysis. **G** and **H** The protein bands of NAMPT, FOXO3A, and MnSOD in the ischemic brain sections were examined by western blot analysis. **I**–**K** The relative expression levels of SIRT1, SIRT2 and SIRT3 were quantified and analysed by using Gel-Pro analyser software. **L**–**N** The relative expression levels of NAMPT, FOXO3A, and MnSOD were quantified and analysed by using Gel-Pro analyser software. Mean values ± SEM (n=3–6). Statistical comparisons were performed as follows: one-way ANOVA with Dunnett’s multiple comparisons test for (C, D, K, L, and N); unpaired t test for (**I**, **J**, **M**, and **K**); ^*^P<0.05, ^**^P<0.01 versus MCAO/R group; ^#^P<0.05, ^##^P<0.01 versus Sham group

Hypoxia-induced autophagy blocks the neuroapoptosis pathway induced by mitochondrial injury through the Hif1α/BNIP3 signalling pathway [19, 20]. We wondered if Hif1α and BNIP3 participated in the mechanisms underlying the regulation of autophagy by GP17. Western blot analysis revealed that GP17 pretreatment significantly increased Hif1α and BNIP3 expression in hippocampal and/or cortical tissues compared with that in MCAO/R rats. Furthermore, RAP pretreatment activated Hif1α in the cortex and BNIP3 in the hippocampus. There were no notable differences in Hif1α and BNIP3 expression in the hippocampus and cortex regions in 3-MA-treated rats compared with rats subjected to MCAO/R alone (Fig. 3A–D).

Our previous study revealed that the NAMPT-SIRT pathway can increase neuronal ischemic tolerance, inhibit neuronal apoptosis and necrosis, and improve energy metabolism under ischemia [13, 14]. In this study, we found that SIRT1/2/3 expression in the hippocampus and/or cortex was markedly activated in ischemic rats compared with sham rats but that the change in SIRT1/2 expression after ischemia was significantly attenuated by GP17 pretreatment (Fig. 3E–F, I–K).

To further explore the effect of GP17 on the downstream proteins regulated by NAMPT-SIRT1/2, we detected the expression of NAMPT, FOXO3A and MnSOD and explored the regulatory effect of GP17 on FOXO3A. We found that rats treated with GP17 exhibited higher expression of NAMPT, MnSOD and FOXO3A than those treated with vehicle in the hippocampus and/or cortex regions (Fig. 3G–H, L–N). These findings implied that GP17 activated the expression of the downstream protein SIRT1/2 through NAMPT, upregulated FOXO3A and MnSOD expression, inhibited mitochondrial oxidative damage, improved energy metabolism, and thereby inhibited neurological damage caused by ischemia.

### GP17 attenuates OGD/R-induced SH-SY5Y cell damage by regulating mitochondrial autophagy in vitro

**Fig. 4:**
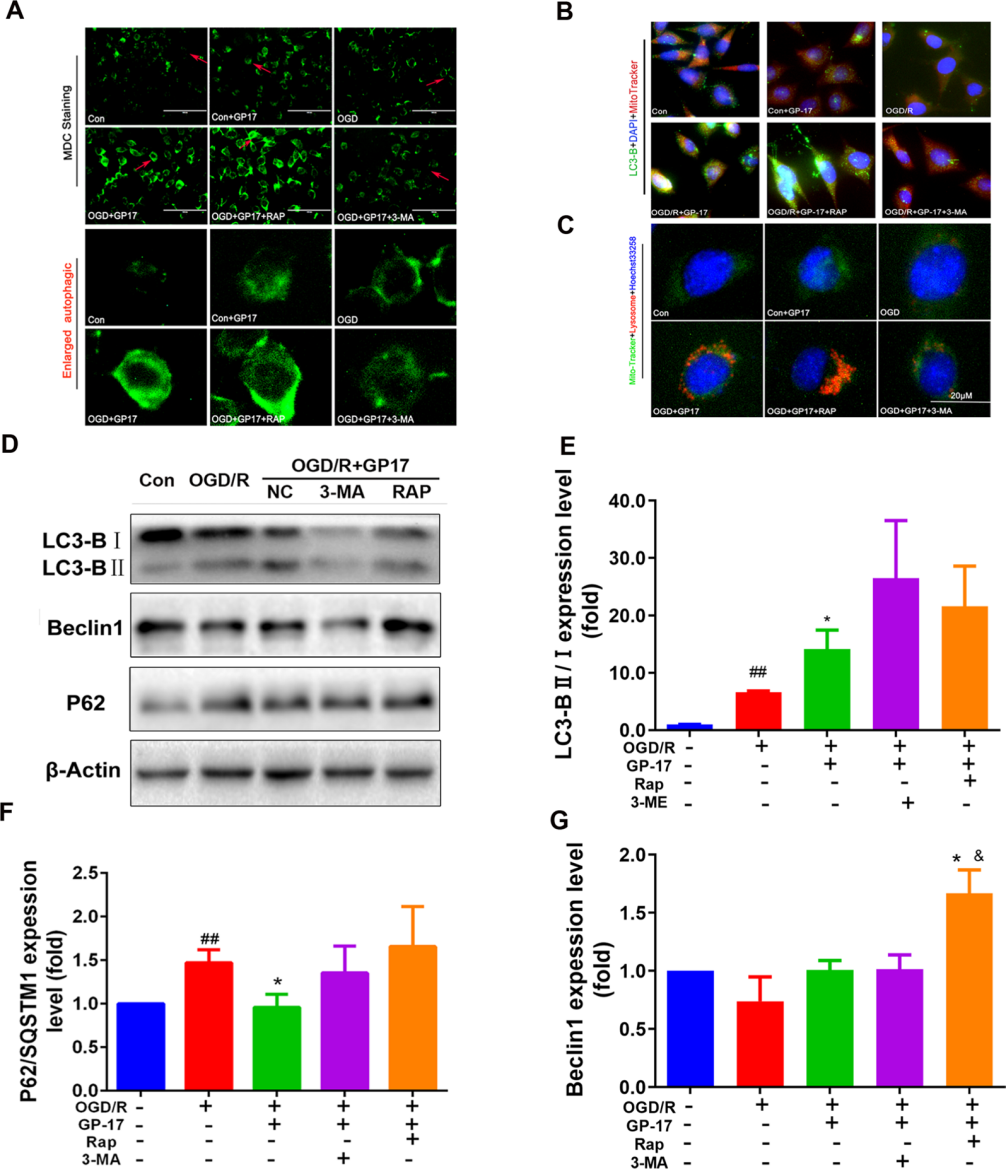
The effects of GP17 on autophagic vesicles, autophagic lysosomes, LC3-B expression and the Beclin1-LC3-P62 signalling pathway in OGD/R-subjected SH-SY5Y cells were partly reversed by the inhibitor 3-MA. **A** MDC staining from all SH-SY5Y cell groups as measured by a fluorescence microscope; scale bar = 100 μm. **B** Triple staining of autophagosome-lysosomes (red), mitochondria (green) and nuclei (blue) in OGD/R-subjected SH-SY5Y cells, as measured by a fluorescence microscope; scale bar = 20 μm. **C** Triple staining of LC3-B protein (green), mitochondria (red) and nuclei (blue) in OGD/R-subjected SH-SY5Y cells, as located and measured by a fluorescence microscope; scale bar, 20 μm. **D** The protein bands of LC3-B, Beclin1 and P62 were examined by western blot analysis in OGD/R-subjected SH-SY5Y cells. **E–G**. The expression levels of Beclin1 and P62, and the ratio LC3-B II and I in OGD/R-subjected SH-SY5Y cells were quantified and analysed by using Gel-Pro analyser software. The data presented as Mean values ± SEM. Statistical comparisons were performed as follows: unpaired t test for (**E**, **F**, and **G**); ^#^P < 0.05, ^##^P < 0.01 versus the control group; ^*^P < 0.05 versus the OGD/R model group; ^&^P < 0.05 versus the OGD/R + GP17 group

To better characterize the effect of GP17 on mitochondrial autophagy regulation and to identify the possible mechanism involved, we first detected autophagic vesicles in OGD/R-subjected SH-SY5Y cells in vitro. GP17 notably increased the number and fluorescence intensity of acidic autophagic vesicles whose formation was induced by OGD/R. To explore whether autophagy was involved in the effect of GP17 on OGD/R-induced SH-SY5Y cell damage, OGD/R-subjected SH-SY5Y cells were treated with RAP and 3-MA in addition to GP17. The results showed that RAP pretreatment further increased the number and fluorescence intensity of acidic autophagic vesicles, while 3-MA almost completely eliminated the enhancement of the number and fluorescence intensity of acidic autophagic vesicles caused by GP17 (Fig. 4A). To examine autophagic lysosomes in vitro, we incubated OGD/R-subjected SH-SY5Y cells with GP17 and examined autophagic lysosomes by immunostaining with Lyso-Tracker Red and MitoTracker Green. As shown in Fig. 4B, GP17 notably increased autophagy in OGD/R-subjected SH-SY5Y cells. Mitophagosome formation was also evaluated according to the colocalization of the autophagy marker LC3-B (green) and the mitochondria marker MitoTracker Red. As shown in Fig. 4C, mitochondrial autophagy was notably increased in GP17-treated cells, as indicated by increased colocalization of green LC3-B and MitoTracker Red, and RAP pretreatment more strongly increased mitochondrial autophagy than GP17. In contrast, mitochondrial autophagy was significantly decreased in GP17-treated cells pretreated with 3-MA. These results suggest that GP17 activates mitochondrial autophagy in OGD/R-subjected SH-SY5Y cells.

We continued to examine the expression of major mitochondrial autophagy signalling pathway-related proteins in SH-SY5Y cells. We found that GP17 increased LC3-B and Beclin1 expression and inhibited p62 expression in OGD/R-subjected SH-SY5Y cells. The upregulation of all proteins except p62 was exacerbated by RAP (Fig. 4D–G).

### SIRT1/2 and Hif1α inhibition abolishes the effect of GP17 on mitochondrial autophagy-related indicators in OGD/R-subjectedSH-SY5Y cells

To assess whether the SIRT1/2 and/or Hif1α signalling pathways were associated with the mitochondrial autophagy-related indicators of GP17, the SIRT1/2/3 inhibitor AGK-7 and the Hif1α inhibitor 2-ME were used to treat SH-SY5Y cells along with GP17. First, we investigated the viability of normal SH-SY5Y cells treated with AGK-7 (0.1, 0.5, 1.0, 5.0 and 10 µM) and 2-ME (1.0, 5.0, 10.0 and 20.0 µM). As shown in Additional file 1: Figure S2A and B, compared with the control treatment, AGK-7 incubation at 0.5–10 µM and 2-ME incubation at 10.0 and 20.0 µM significantly reduced the viability of SH-SY5Y cells. We selected 0.1 µM AGK-7 and 2.5 µM 2-ME to further verify the molecular mechanism by which GP17 regulates mitochondrial autophagy.

**Fig. 5:**
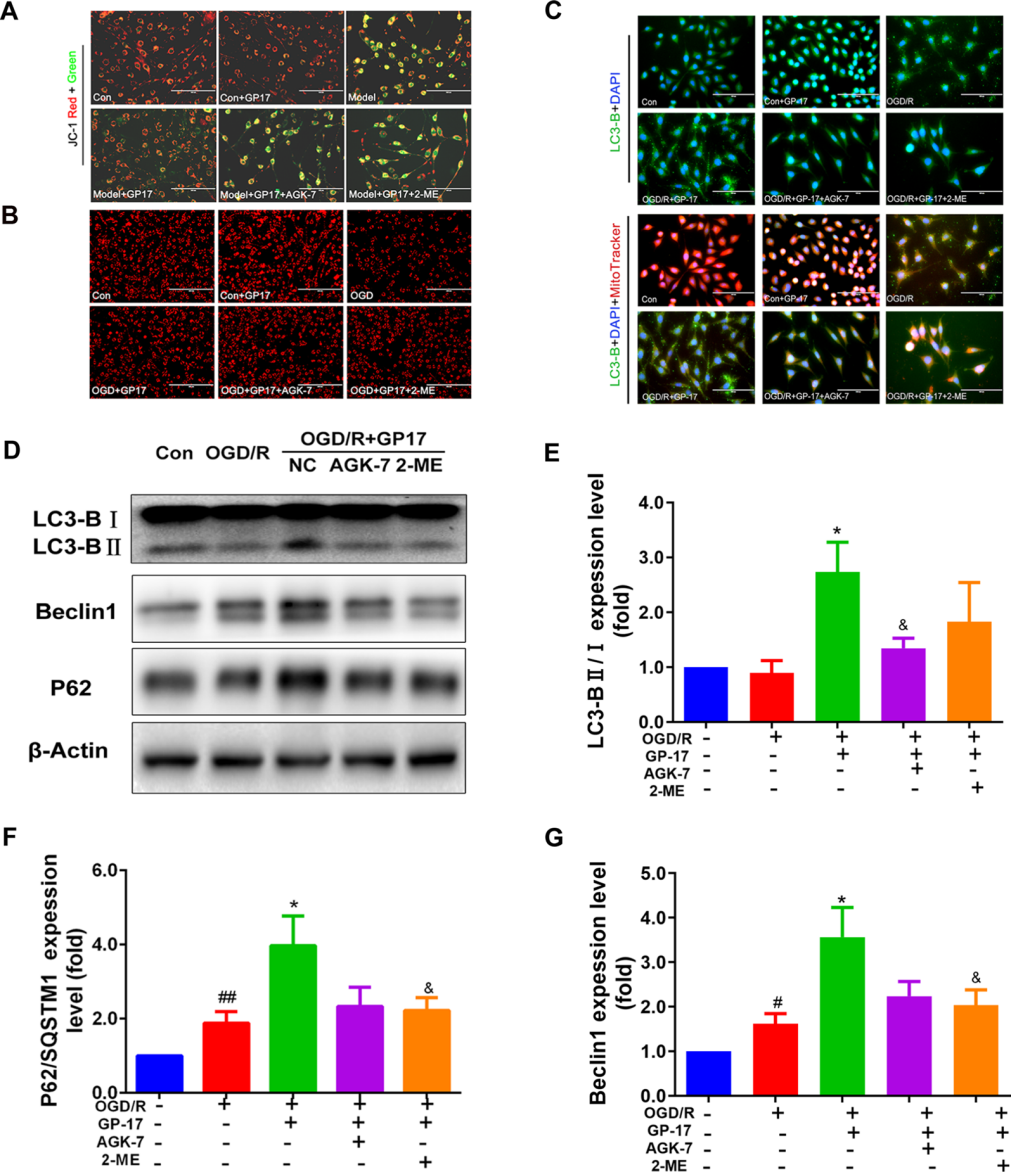
Effects of GP17 on mitochondria-related indicators and autophagy. The Beclin1-LC3-P62 signalling pathway in OGD-induced SH-SY5Y cells was suppressed by the inhibitors AGK-7 and 2-ME. **A** Mitochondrial fluorescence images of GP17 to assess the MMP in OGD/R-subjected SH-SY5Y cells, as measured by JC-1 assay with a fluorescence microscope; scale bar, 100 μm. **B** Representative images of mitochondria stained with MitoTracker Red-CMXRos obtained with a fluorescence microscope; scale bar, 200 μm. **C** Triple staining of LC3-B protein (green), mitochondria (red) and nuclei (blue) in OGD/R-subjected SH-SY5Y cells, as located and measured by a fluorescence microscope; scale bar = 20 μm. **D** The protein bands of LC3, Beclin1 and P62 were examined by western blot analysis in OGD/R-subjected SH-SY5Y cells. **E–G** The expression levels of Beclin1 and P62, and the ratio LC3-B II and I in OGD/R-subjected SH-SY5Y cells were quantified and analysed by using Gel-Pro analyser software. The data presented as Mean values ± SEM. Statistical comparisons were performed as follows: unpaired t test for (**E**, **F**, and **G**); ^#^P < 0.05, ^##^P < 0.01 versus the control group; ^*^P < 0.05 versus the OGD/R model group; ^&^P < 0.05 versus the OGD/R +  1 group

We next evaluated the mitochondrial membrane potential (MMP) and viability of SH-SY5Y cells upon challenge with OGD/R and GP17 or AGK-7 and 2-ME treatment. Figure 5A, B show that either AGK-7 or 2-ME decreased the MMP and mitochondrial viability of GP17- and OGD/R-cotreated SH-SY5Y cells. To determine the effect of GP17 on mitochondrial function, we evaluated oxidative phosphorylation and glycolysis by measuring the oxygen consumption rate (OCR) and extracellular acidification rate (ECAR), respectively, in a Seahorse assay. Additional file 1: Figure S3A–D shows that the OCR and ECAR in the GP17- and OGD/R-cotreated groups were significantly higher than those in the OGD/R group. However, AGK-7 and 2-ME abolished the beneficial effect of GP17 on mitochondrial function in OGD/R-subjected SH-SY5Y cells.

To further assess whether the SIRT1/2 and/or Hif1α signalling pathways were involved in the regulation of mitochondrial autophagy by GP17, AGK-7 and 2-ME were used to treat SH-SY5Y cells along with GP17. Figure 5D shows that both AGK-7 and 2-ME significantly inhibited the colocalization of GFP-LC3-B and MitoTracker Red enhanced by GP17. The western blot results also confirmed that AGK-7 and/or 2-ME decreased LC3-B, Beclin1 and p62 expression in the GP17- and OGD/R-cotreated group (Fig. 5E–G). These data suggest that GP17 can ameliorate ischemia-/hypoxia-induced neuronal injury by regulating mitochondrial autophagy and that this effect is closely related to the SIRT1/2 and Hif1α pathways.

In addition, GP17 incubation for 24 h significantly attenuated OGD/R-induced apoptosis. However, the inhibitory effect of GP17 on apoptosis was reduced in the AGK-7 and 2-ME treatment groups compared with the OGD/R + GP17 group, suggesting that the reduction in ischemia-induced apoptosis by GP17 may occur through NAMPT-regulated downstream SIRT1/2 signalling and the Hif1α pathway. These results were consistent with the in vivo results (Additional file 1: Figure S2C).

### SIRT1/2 and Hif1α inhibition abolishes the effects of GP17 on the Hif1α-BNIP3 and SIRT1/2-FOXO3A signalling pathways in OGD/R-subjected SH-SY5Y cells

**Fig. 6:**
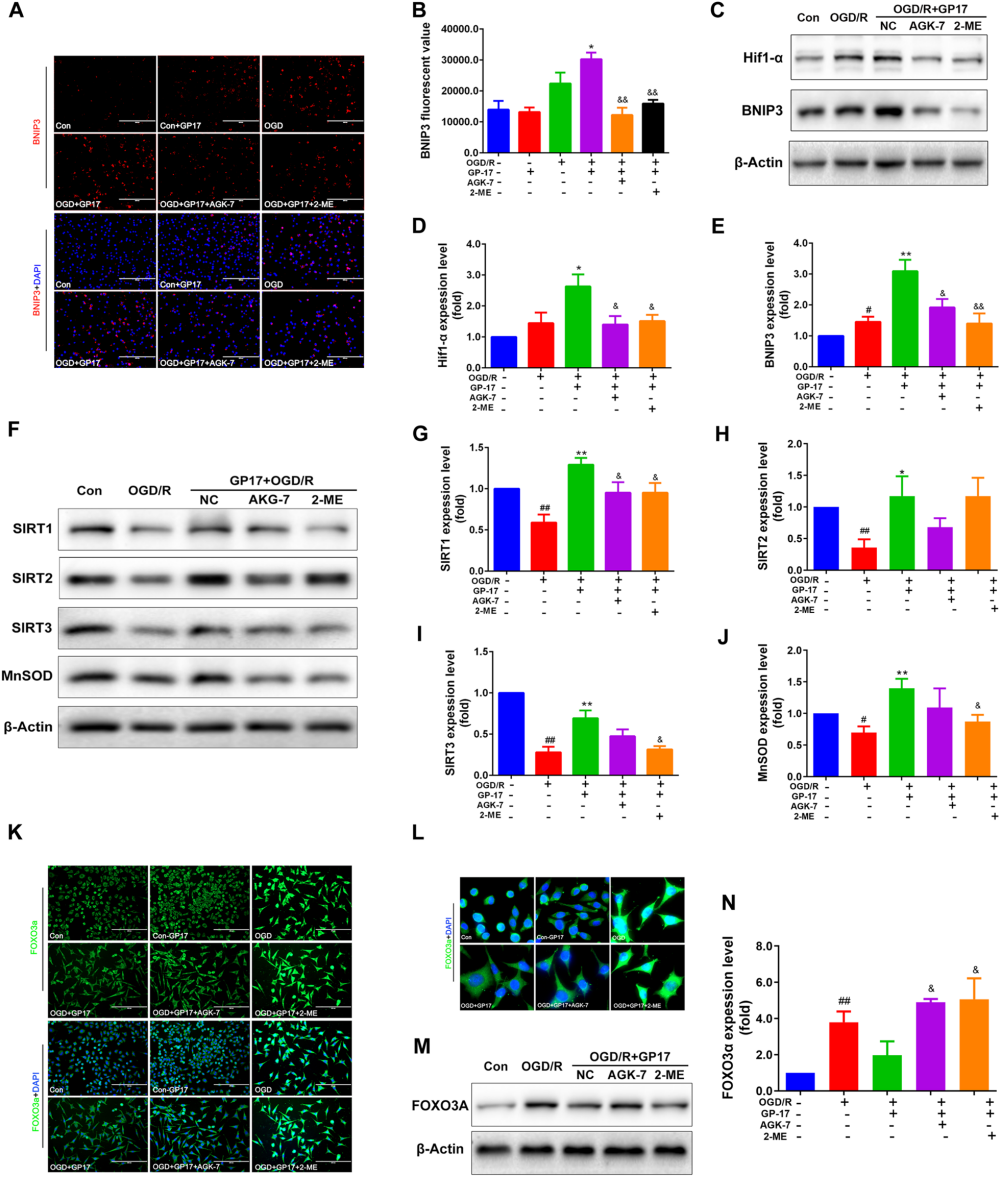
Effects of GP17 on the Hif1α-BNIP3 and SIRT1/2/3-FOXO3A signalling pathways in OGD/R-subjected SH-SY5Y cells. These effects were attenuated by the inhibitors AGK-7 and 2-ME. **A** Double staining of the BNIP3 protein (red) with the nuclei (blue) in OGD/R-subjected SH-SY5Y cells, as located and measured by a fluorescence microscope; scale bar = 200 μm. **B** Statistical analysis of BNIP3 fluorescence via ImageJ software. **C** The protein bands of Hif-1α and BNIP3 were examined by western blot analysis in OGD/R-subjected SH-SY5Y cells. **D**, **E** The relative expression levels of Hif-1α and BNIP3 in OGD/R-subjected SH-SY5Y cells were quantified and analysed by using Gel-Pro analyser software. The data are presented as the mean values ± SDs (n = 3–6). F. The protein bands of SIRT1/2/3 and MnSOD were examined by western blot analysis in OGD/R-subjected SH-SY5Y cells. **G–J** The relative expression levels of SIRT1/2/3 and MnSOD in OGD/R-subjected SH-SY5Y cells were quantified and analysed by using Gel-Pro analyser software. The data are presented as the mean values ± SDs (n = 3). K. Double staining of the FOXO3A protein (green) with the nuclei (blue) in OGD/R-subjected SH-SY5Y cells, as located and measured by a fluorescence microscope; scale bar = 100 μm. **L** Enlarged images of FOXO3A protein. **M** The relative expression levels of FOXO3A in OGD/R-subjected SH-SY5Y cells were quantified and analysed by using Gel-Pro analyser software. N. The protein bands of FOXO3A were examined by western blot analysis in OGD/R-subjected SH-SY5Y cells. The data presented as Mean values ± SEM. Statistical comparisons were performed as follows: unpaired t test for (**B**, **D**, **E**, **G**, **H**, **I**, **J** and **N**); ^#^P < 0.05, ^##^P < 0.01 versus the control group; ^*^P < 0.05, ^**^P < 0.01 versus the OGD/R model group; ^&^P < 0.05, ^&&^P < 0.01 versus the OGD/R + GP17 group

GP17 regulates mitochondrial autophagy through activation of the Hif1α and/or SIRT1/2 signalling pathways. The regulation of the Hif1α-BNIP3 and SIRT1/2-FOXO3A pathways by GP17 was explored using immunofluorescence and western blotting, and the potential mechanism was verified with the inhibitors AGK-7 and 2-ME. Immunofluorescence analysis revealed that GP17 significantly increased BNIP3 expression in OGD/R-subjected SH-SY5Y cells compared with the expression in the cells subjected to OGD/R alone. However, AGK-7 and 2-ME pretreatment reversed the change in BNIP3 expression (Fig. 6A, B). Similarly, both AGK-7 and 2-ME significantly inhibited the Hif1α and BNIP3 protein expression induced by GP17 (Fig. 6C–E).

In an in vivo study, we found that GP17 pretreatment markedly increased SIRT1/2/3 expression in the hippocampus and/or cortex regions compared with that in MCAO/R rats. Here, we also found that GP17 markedly increased SIRT1/2/3 and MnSOD expression in OGD/R-subjected SH-SY5Y cells compared with that in the cells subjected to OGD/R alone. AGK-7 significantly inhibited the increases in the expression of these proteins induced by GP17 (Fig. 6F–J). Notably, the trend of inhibition of SIRT1/3, but not SIRT2, after 2-ME treatment was obvious. Combined with the results of in vivo experiments, these findings indicate that GP17 can regulate the mitochondrial autophagy-related Hif1α-BNIP3 pathway through NAMPT-SIRT1, exerting a neuroprotective effect in the process of hypoxia-ischemia.

To further determine the relationship between GP17-mediated regulation of FOXO3A and the SIRT1/Hif1α/BNIP3 pathway, we detected the expression of FOXO3A in vitro using immunofluorescence and western blotting upon stimulation with GP17, AGK-7 and 2-ME. Figure 6K, L show that cells treated with GP17 exhibited a significantly lower fluorescence intensity of FOXO3A than those treated with vehicle. However, the trend of decreased FOXO3A fluorescence was reversed after 2-ME treatment. The western blot results showed that both AGK-7 and 2-ME attenuated the GP17-mediated reduction in FOXO3A protein expression, indicating that Hif1α and FOXO3A jointly participate in the downstream processes mediated by SIRT1 (Fig. 6M, N).

## Discussion

GP17 is a dammarane-type 20-(S)-protopanaxadiol-type saponin that mainly exists in the stems and leaves of *Panax notoginseng* and *Gynostemma pentaphyllum* and has significant pharmacological activity. Our previous research found that GP17 can significantly inhibit cognitive damage and alleviate MPTP- and glutamate-induced neuronal oxidative damage, indicating that GP17 has neuroprotective effects [17, 18]. In this study, we first conducted in vivo experiments and found that GP17 administration significantly ameliorated neurological dysfunction induced by MCAO/R, reduced the cerebral infarct volume, alleviated the pathological changes in brain tissue caused by ischemia, and inhibited oxidative damage and inflammatory responses during acute cerebral ischemia. However, the molecular mechanisms of the neuroprotective effect of GP17 against ischemic brain injury and the inhibition of mitochondrial damage were still unclear.

In the process of cerebral ischemia, regulation of mitochondrial autophagy can reduce neuronal apoptosis induced by cerebral infarction and produce neuroprotective effects [6]. In this study, we found that GP17 administration regulated the protein expression of Beclin1-LC3-P62 in ischemic brain regions to varying degrees in the MCAO/R rat model, consistent with the findings in the group treated with the autophagy inducer RAP. In the OGD/R cell model, we found that the labelling of autophagic vacuoles, autophagic fluorescent puncta and autophagic lysosomes in the cells was significantly increased after GP17 treatment; in addition, the expression of intracellular Beclin1-LC3-P62 proteins was elevated. However, the above changes were reversed by the inhibitor 3-MA. These results are consistent with those in relevant reports [16, 17, 21]. These in vitro and in vivo results suggest that GP17 can regulate mitochondrial autophagy. However, under GP17 + RAP cotreatment, the excessive activation of autophagy did not increase the inhibitory effect on apoptosis. Combined with the results of rat experiments in vivo, the partial protective effects in both the inhibitor and inducer groups suggested that maintaining appropriate levels of autophagy and inhibiting excessive levels of autophagy in the acute phase of ischemia may alleviate early ischemic brain damage.

Hif1α is a hypoxia-inducing factor that plays a key role in tissue adaptation to ischemia/hypoxia [19, 22]. However, under prolonged hypoxic conditions, Hif1α overexpression regulates mitochondrial respiration, resulting in increased levels of ROS and apoptosis [23–25]. BNIP3 is a mitochondrial outer membrane protein with very low levels of expression under normal physiological conditions, but in hypoxic environments, activated Hif1α upregulates the expression of BNIP3, causing Beclin1 to dissociate from Bcl-XL and Bcl-2 and thereby releasing Beclin1[6, 20]. Beclin1 can promote mitochondrial autophagy by triggering translocation of the E3 ubiquitin ligase Parkin to mitochondria or by directly binding to LC3-B on autophagosomes [6]. Recent studies have found that autophagy activation can be mediated through the Hif1α/BNIP3 pathway. Selective activation of appropriate levels of mitochondrial autophagy clears damaged mitochondria, blocks the release of CytC and the activation of Caspase-9/Caspase-3, stabilizes MMP, and increases the Bcl-2/Bax ratio to block the apoptosis pathway induced by mitochondrial injury, thereby protecting against cerebral ischemic neuronal damage [6, 26]. Our findings are consistent with related reports that MCAO/R induces upregulation of Hif1α and BNIP3, suggesting that GP17 may regulate the Hif1α-BNIP3 pathway to mediate autophagy in the acute phase of cerebral ischemia. To verify the above experimental results, we treated OGD/R-subjected SH-SY5Y cells with 2-ME, a specific inhibitor of Hif1α. We found that GP17 treatment activated the Hif1α-BNIP3 pathway but that 2-ME treatment blocked the upregulation of Hif1α; the transcript expression level of BNIP3 was also reduced.

The SIRTUIN protein family is a class of NAD^+^-dependent protein deacetylases and ADP ribosyltransferases with a highly conserved core region [27–29]. Members of the mammalian SIRT protein family can interact with p53, FOXO, PGC-1α, NF-κB, Ku70 and other proteins to regulate the cellular stress response, thereby affecting biological processes such as cell metabolism, ageing and apoptosis [27, 28, 30]. GP17 is an important active substance in PNGL. In vivo, we found that GP17 regulates the levels of the protein SIRT1/2/3 downstream of NAMPT, mainly SIRT1; activates transcription factors related to antioxidant and inflammatory responses; and improves nerve resistance to ischemia. This finding suggests that GP17 can regulate NAMPT-mediated downstream pathways, and improve neuronal viability.

To further explore the regulation of the SIRT1/2/3 pathway by GP17, we used the specific inhibitor AGK-7 to study the SH-SY5Y cell model subjected to OGD/R. The results showed that OGD/R decreased the expression of SIRT1/2/3, while GP17 treatment significantly increased the protein expression levels of SIRT1, SIRT2 and SIRT3. However, treatment with the inhibitor AGK-7 significantly reversed the changes in the expression of SIRT1 and SIRT3, especially the change in the expression of SIRT1. The comprehensive in vitro and in vivo results suggest that the protective effect of GP17 in the process of hypoxia-ischemia may be related mainly to regulation of SIRT1. We also found that the inhibitor AGK-7 attenuated the upregulation of Hif1α caused by GP17. Combined with related reports, these findings suggest that SIRT1, upstream of Hif1α, and BNIP3 are involved in the regulation of mitochondrial autophagy under ischemia.

In addition, treatment with the inhibitors AGK-7 and 2-ME blocked the GP17-mediated regulation of the SIRT1/2/3 and Hif1α-BNIP3 molecular pathways. Furthermore, the regulation of autophagic puncta and Beclin1 and LC3-B expression by GP17 was significantly reversed by the inhibitor 2-ME and partially reversed by the inhibitor AGK-7. As the changes in autophagy and mitochondrial regulation were reversed, the neuroprotective effect of GP17 was partially blocked. These results indicate that GP17 can alleviate ischemic injury, reduce mitochondrial dysfunction, increase the number of mitochondria, and improve mitochondrial viability. This protective effect is achieved through mitochondrial autophagy mediated by the SIRT1/2/3 and Hif1α-BNIP3 pathways.

FOXO3A is a human protein encoded by the FoxO3 gene whose family includes FoxO1, FoxO3, FoxO4 and FoxO6. Recent research has shown that FOXO3A can regulate cell differentiation and proliferation, improve metabolism, inhibit apoptosis, repair damaged DNA, and regulate oxidative stress through the PI3K-AKT-FOXO3A, SIRT1-FOXO3A, AMPK-FOXO3A-MnSOD and other pathways [31–35]. In a previous study, we found that PNGL has a protective effect through the SIRT1-FOXO3A pathway, and the current study revealed that GP17 can regulate FOXO3A acetylation in the cytoplasm through SIRT1/2 in in vivo and in vitro models, increase the binding of FOXO3A to DNA, and enhance the expression of FOXO3A target genes and promote cell survival. This mechanism may be the common molecular mechanism of PNGL components against ischemic injury. In addition, intracellular MnSOD protein expression was significantly increased after GP17 treatment in the present study, and MnSOD expression was reversed after treatment with the inhibitor AGK-7, indicating that SIRT1/3 is an important upstream regulatory molecule.

FOXO3A is at the intersection of multiple signalling pathways in oxidative stress. FOXO3A induces autophagy by inducing the expression of autophagy-related genes, such as LC3, BNIP3, Beclin1, and Atg12, but excessive autophagy can induce apoptosis [31, 36, 37]. Previous studies have found that SIRT1/2/3 (mainly SIRT1) may interact with Hif1α upstream of Hif1α during GP17 regulation of ischemic injury [38]. We further found that GP17 treatment significantly reduced the intracellular FOXO3A level induced by OGD/R but that the downregulation of FOXO3A by GP17 was reversed by 2-ME, a specific inhibitor of Hif1α; these findings suggest that the regulation of SIRT1-FOXO3A involves the Hif1α-BNIP3 pathway and that the two regulatory effects are opposite. They further imply that GP17 can inhibit the overactivation of the Hif1α-BNIP3 pathway induced by ischemia and hypoxia, allowing the two to participate in the processes of autophagy homeostasis.

**Fig. 7:**
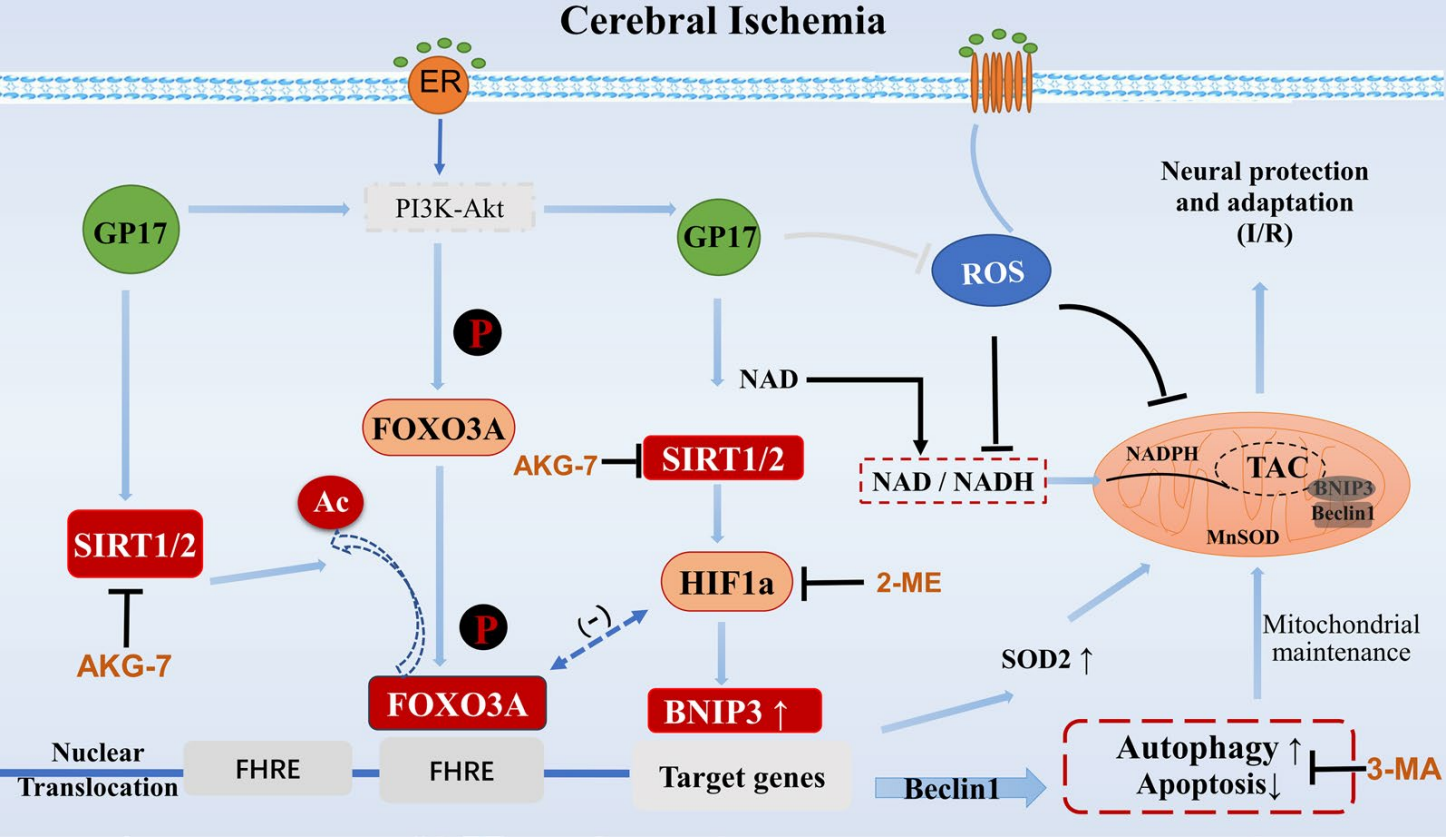
Effects and mechanisms of GP17 against cerebral ischemic injury via mitochondrial autophagy regulation mediated by the SIRT1/2/3-FOXO3A/Hif1a-BNIP3 signalling pathways. These effects inhibit mitochondrial damage, improve energy metabolism, and alleviate neuronal apoptosis

Taken together, the findings of this study confirm the preventive and therapeutic effects of GP17 on ischemic stroke. We explored and analysed the effects and molecular mechanisms by which GP17 inhibits mitochondrial damage and ischemia-induced neuroapoptosis. The mechanism involves, in part, SIRT1-FOXO3A/Hif1a-BNIP3-mediated mitochondrial autophagy, which regulates the downstream signalling pathways of PGC-1α, SOD1, and SOD2; inhibits mitochondrial damage; alleviates oxidative stress and apoptosis induced by ischemia and hypoxia; improves neuroviability; and has a preventive effect against ischemic stroke (Fig. 7). Therefore, this study elucidates the effects and molecular mechanisms of GP17 in ameliorating ischemic injury and shows that GP17 is a very promising compound for the development of new therapeutic agents for ischemic stroke.

## Materials and methods

### Materials

GP17 (molecular weight = 947.154; purity > 98%) was purchased from Beijing Beina Chuanglian Biotechnology Research Institute (Beijing, China). The positive control drug NBP was obtained from CSPC NBP Pharmaceutical Co., Ltd. Triphenyltetrazolium chloride (TTC) was purchased from Sigma–Aldrich (MO, United States). Primary antibodies against p62/SQSTM1, LC3-B, BNIP3, NAMPT, SIRT1, SIRT2, MnSOD, PGC-1α, FOXO3 and p-FOXO3 were obtained from Abcam (Cambridge, UK). Primary antibodies against Hif1α and Beclin1 were obtained from Proteintech (Wuhan, China). A primary antibody against SIRT3 was obtained from Cell Signaling Technology (MA, USA). The inducer RAP and the inhibitors 3-MA and 2-ME were obtained from MedChemExpress (New Jersey, USA). The inhibitor AGK-7 was obtained from Abcam (Cambridge, UK). ELISA kits for IL-6, TNF–α, MCP-1, T-AOC and 4-HNE were acquired from HaiTai TongDa Sci Tech, Ltd. (Beijing, China).

### Animals

Male Sprague–Dawley rats (obtained from Beijing Vital Lihua Experimental Animals Co., Ltd.) (weighing 220 ~ 240 g) were used in this study. All operations and treatments were required to conform to the Declaration of Helsinki and the “3Rs” principle. The experimental protocol was approved in accordance with the guidelines of the Laboratory Animal Ethics Committee (Permit Number: SYXK 2017-0020). All rats were housed in ventilated cages with a 12-hour light/dark cycle at 20 ~ 25 °C in a temperature-controlled room with free access to food and water.

### MCAO surgery

The Sprague–Dawley rats were anaesthetized by i.p. injection with Zoletil 50 (10 ~ 15 mg/kg) and subjected to the MCAO procedure. A rat model of cerebral ischemia/reperfusion (I/R) was established using the suture occlusion technique described in our previous study [39]. The sham-operated rats underwent the same procedure except that the sutures were not inserted into the internal carotid artery. The rats’ body temperature was maintained at 37 ± 0.5 °C using a heating pad (Sunbeam, United States). The grouping of animals was conducted in a blinded manner; the researchers did not know which group each of the animals was assigned to.

### Drug treatment

The drug was dissolved in 0.9% normal saline prior to administration. The drug was administered by i.p. injection. To select the drug doses, GP17 (10 mg/kg and 20 mg/kg) and NBP (positive control drug, 20 mg/kg) were administered for 7 days before MCAO surgery. To detect mitochondrial autophagy, rats were divided into 5 groups: the sham group, the MCAO/R group, the GP17 (20 mg/kg) + MCAO/R group, the RAP (2 mg/kg) + MCAO/R group and the 3-MA (3 mg/kg) + MCAO/R group. The sham and MCAO/R groups were given 0.9% normal saline solution by i.p. injection daily.

### Neurological scoring

Neurological behaviour was investigated at 24 h after I/R by two blinded investigators using Zea Longa scores [40, 41] and Bederson scores [42], as previously published. Higher scores represented more severe neurological deficits.

### TTC staining

TTC staining was conducted 24 h after I/R to determine the infarct volume and was performed as previously described [12]. Two-millimetre coronal slices were obtained to calculate the infarct volume. Brain slices were stained with 2% TTC at 37 °C for 20 min and fixed overnight in 4% paraformaldehyde. The cerebral infarct area on each slice was quantified by using ImageJ 1.44p software (National Institutes of Health, Bethesda, MD, USA).

### Haematoxylin and eosin (H&E) staining

H&E staining was performed according to previously described methods [12, 43]. Intact brains were prepared, and pathological paraffin Sect. (5-µm thick) were made. The staining images were acquired using a pathological scanner and analysis system (Aperio CS2, Leica, Wetzlar, Germany).

### OGD/R and drug treatment

The OGD/R method was described in our previous study [44]. Briefly, SH-SY5Y cells were cultured in glucose-free Locke’s medium in an anaerobic chamber (Type C; Coy Laboratory Products, Inc., Grasslake, MI, United States) for 4 h. Then, the cells were removed from the anaerobic chamber to a normoxic environment, and the medium was replaced with normal medium for 24 h. In the GP17-treated group, SH-SY5Y cells subjected to ODG/R were pretreated with GP17 (6.25 µM) for 24 h. In the inhibitor-treated group, the cells were preincubated with 100 nM AGK-7 and 1.0 µM 2-ME for 1 h prior to treatment with GP17.

### Determination of MMP

MMP was assessed using a JC-1 MMP assay kit (C2006, Beyotime, Shanghai, China) as we have described previously [14]. Briefly, SH-SY5Y cells were washed twice with FBS-free DMEM, incubated with JC-1 (200 µM) for 20 min at 37 °C, and then washed with DMEM to remove the excess JC-1. Fluorescence images of JC-1 in the cells were obtained using a fluorescence microscope (EVOS M5000, Thermo Fisher Scientific, USA). MMP was calculated according to the ratio of red to green fluorescence.

### Assessment of mitochondrial viability

After incubation and treatments, mitochondrial viability was evaluated using our previously described method [14]. SH-SY5Y cells were washed twice with DMEM, incubated with the probe MitoTracker Red CMXRos (50 nM) for 45 min at 37 °C, and washed with DMEM. Fluorescence images of mitochondrial viability in cells were obtained using fluorescence microscopy (EVOS M5000, Thermo Fisher Scientific, USA).

## Immunofluorescence staining

Immunofluorescence staining was performed using our previously described method [44, 45]. Primary antibodies against LC3-B, BNIP3 and FOXO3A were used for the experiment. Images were obtained through at least three random visual fields from three separate sections of each sample by using a fluorescence microscope (Carl Zeiss, Germany).

### Enzyme-linked immunosorbent assay (ELISA)

ELISA kits were used according to the manufacturer’s instructions and a previously described method [12, 46] to quantify IL-6, TNF-α, MCP-1, T-AOC and 4-HNE in serum.

### Transmission electron microscopy

Transmission electron microscopic (TEM) analyses of the accumulation of autophagosomes on ischemic cortex induced by MCAO/R were performed as previously described [13]. Brain sections were fixed with electron microscope fixative solution for 2 ~ 4 h and then with 1% osmium acid 0.1 M phosphate buffer (PH7.4) fixed at room temperature (20 °C) for 2 h. Then, dehydrated in an ascending ethanol series, and embedded in epoxy resin. Ultrathin Sect. (0.1 mm) were cut, stained with uranyl acetate and lead citrate, and then observed under a TEM (HT7700, HITACHI, Tokyo, Japan).

### Western blot analysis

Western blot analysis was performed according to our previously described method [14, 43]. Based on the standard operating procedures, total proteins were extracted with lysate, and equal amounts of proteins were separated by 10% SDS polyacrylamide gel electrophoresis. The proteins were transferred to nitrocellulose membranes, and the membranes were blocked before being incubated overnight at 4 °C with the appropriate primary antibodies against the following proteins: p62/SQSTM1 (ab56416, 1:1000), LC3-B (ab48394, 1:1000), Hif1α (20960-1-AP, 1:1000), BNIP3 (ab239976, 1:1000), Beclin1 (11306-1-AP, 1:1000), NAMPT (ab236873, 1:1000), SIRT1 (ab189494, 1:1000), SIRT2 (ab211033, 1:2000), SIRT3 (cst5490, 1:1000), FOXO3A (ab109629, 1:3000), p-FOXO3A (ab52857, 1:1000), MnSOD (ab137037, 1:5000), PGC-1a (ab188102, 1:5000) and β-actin (EXP0041F, 1:3000).

### Statistical analysis

GraphPad software was used for statistical analysis. All other data were analyzed using one-way ANOVA followed by the least significant difference (LSD) or Bonferroni’s method and unpaired t test, and p < 0.05 was considered statistically significant.

## Supplementary Information

**Additional file 1: Fig S1.** Effectsof GP17 on inflammation, oxidative stress, and apoptosis. The serum IL-6 (A)、TNF-α (B)、MCP-1(C)、T-AOC (D) and 4-HNE (E)concentrations in MCAO/R rats, determined by ELISA and specificassay kit (n = 4-7 in each group). F. TheTUNEL staining in OGD/R-induced SH-SY5Y cells, measuredby a fluorescencemicroscope, scale bar=200 μm. Mean values ± SEM;^*^P＜0.05, ^**^P＜0.01 versus MCAO/Rgroup; ^#^P＜0.05, ^##^P＜0.01, versus Shamgroup.** Fig. S2.** Effectsof AGK-7, 2-ME and GP17 on SH-SY5Y cell viability and apoptosis. A and B. Thetoxic effect of AGK-7 and 2-ME treatment on SH-SY5Y cells. The cell viabilitymeasured at 490 nM by using the MTT assay (n = 6). C. The TUNEL staining in OGD/R-induced SH-SY5Y cells, measured by afluorescence microscope, scale bar=200 μm. The data presented as Mean values ±SEM. ^#^P < 0.01, ^##^P < 0.01 versus the control group. **Fig. S3.** Effectsof GP17 on the cell energy metabolism in OGD/R-induced SH-SY5Y cells, whichpartly reversed by the inhibitors AGK-7 and 2-ME. A. Effects of GP17 on themitochondrial respiratory and glycolysis OCR levels in OGD/R-induced SH-SY5Ycells, detected by the Seahorse XFp Extracellular Flux Analyzer. B. Effects ofGP17 on the mitochondrial respiratory and glycolysis OCR in OGD/R-inducedSH-SY5Y cells, detected by the Seahorse XFP Extracellular Flux Analyzer. C and D.The statistics values of OCR and ECAR levels presented by the Seahorse XFPExtracellular Flux Analyzer. The data, presented as Mean values ± SEM (n=3-4).

## Data Availability

All data generated or analysed during this study are included in this article (and its Additional files).

## Abbreviations

CIRI: Cerebral ischemia/reperfusion injury
GP17: Gypenoside XVII
MCAO/R: Middle cerebral artery occlusion/reperfusion
OGD/R: Oxygen-glucose deprivation/reoxygenation
NAMPT: Nicotinamide phosphoribosyltransferase
NBP: dl-3-n-butylphthalide
TTC: Triphenyltetrazolium chloride
MMP: Mitochondrial membrane potential
