# Supplementary material for: Protective effects of Gypenoside XVII against cerebral ischemia/reperfusion injury via SIRT1-FOXO3A- and Hif1a-BNIP3-mediated mitochondrial autophagy
Source: J Transl Med. 2022 Dec 27;20:622. doi: 10.1186/s12967-022-03830-9 (PMC9793669; doi:10.1186/s12967-022-03830-9)
Supplement: Supplementary file 1 — Additional file 1: Fig S1. Effectsof GP17 on inflammation, oxidative stress, and apoptosis. The serum IL-6 (A)、TNF-α (B)、MCP-1(C)、T-AOC (D) and 4-HNE (E)concentrations in MCAO/R rats, determined by ELISA and specificassay kit (n = 4-7 in each group). F. TheTUNEL staining in OGD/R-induced SH-SY5Y cells, measuredby a fluorescencemicroscope, scale bar=200 μm. Mean values ± SEM;*P＜0.05, **P＜0.01 versus MCAO/Rgroup; #P＜0.05, ##P＜0.01, versus Shamgroup. Fig. S2. Effectsof AGK-7, 2-ME and GP17 on SH-SY5Y cell viability and apoptosis. A and B. Thetoxic effect of AGK-7 and 2-ME treatment on SH-SY5Y cells. The cell viabilitymeasured at 490 nM by using the MTT assay (n = 6). C. The TUNEL staining in OGD/R-induced SH-SY5Y cells, measured by afluorescence microscope, scale bar=200 μm. The data presented as Mean values ±SEM. #P < 0.01, ##P < 0.01 versus the control group. Fig. S3. Effectsof GP17 on the cell energy metabolism in OGD/R-induced SH-SY5Y cells, whichpartly reversed by the inhibitors AGK-7 and 2-ME. A. Effects of GP17 on themitochondrial respiratory and glycolysis OCR levels in OGD/R-induced SH-SY5Ycells, detected by the Seahorse XFp Extracellular Flux Analyzer. B. Effects ofGP17 on the mitochondrial respiratory and glycolysis OCR in OGD/R-inducedSH-SY5Y cells, detected by the Seahorse XFP Extracellular Flux Analyzer. C and D.The statistics values of OCR and ECAR levels presented by the Seahorse XFPExtracellular Flux Analyzer. The data, presented as Mean values ± SEM (n=3-4). [file 12967_2022_3830_MOESM1_ESM.docx]

**
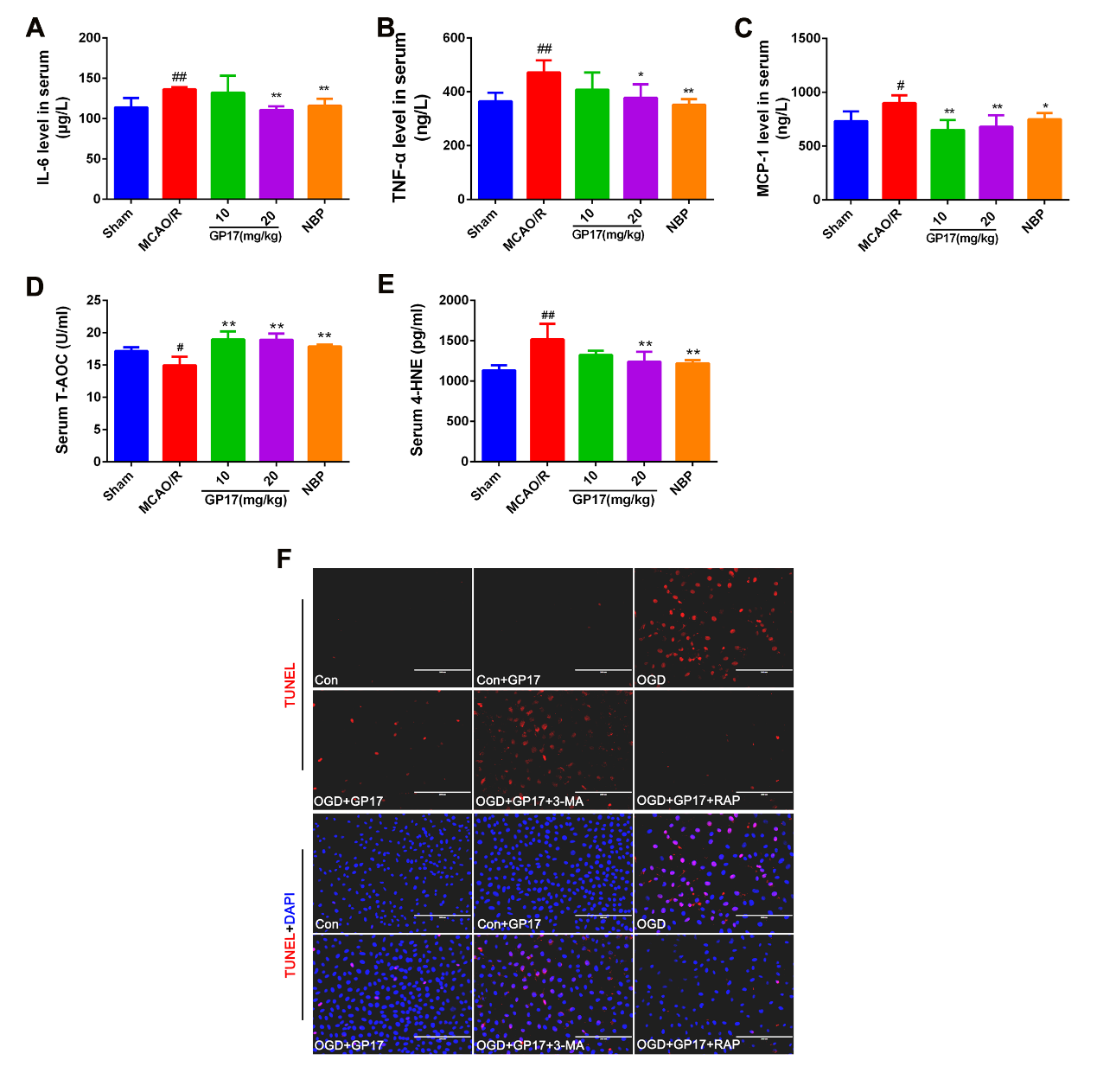
**

**Fig. S1.** Effects of GP17 on inflammation, oxidative stress, and apoptosis. The serum IL-6 (A)、TNF-α (B)、MCP-1(C)、T-AOC (D) and 4-HNE (E) concentrations in MCAO/R rats, determined by ELISA and specific assay kit (n = 4-7 in each group). F. The TUNEL staining in OGD/R-induced SH-SY5Y cells, measured by a fluorescence microscope, scale bar=200 μm. Mean values ± SEM; ^*^P＜0.05, ^**^P＜0.01 versus MCAO/R group; ^#^P＜0.05, ^##^P＜0.01, versus Sham group.

**
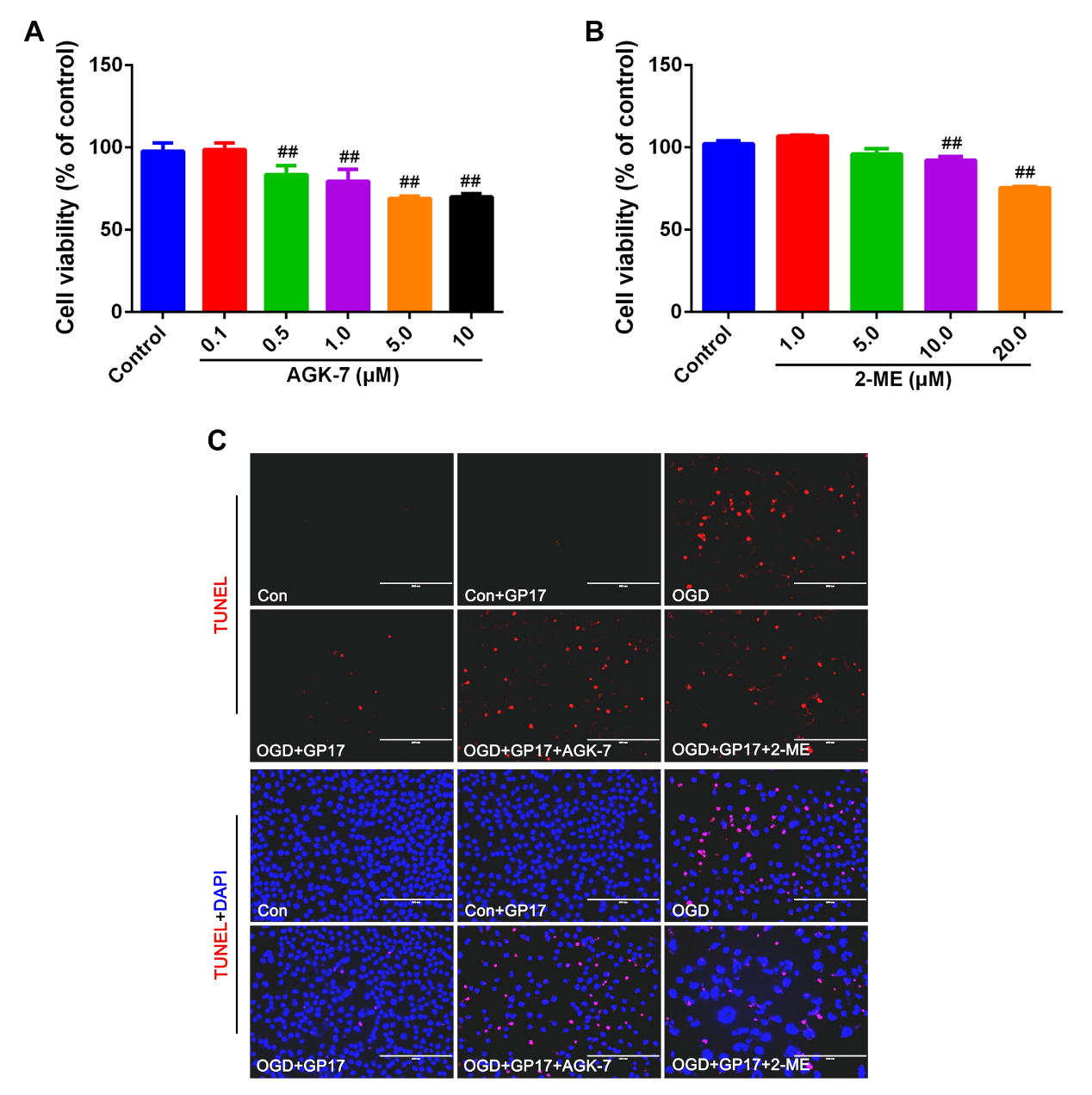
**

**Fig. S2.** Effects of AGK-7, 2-ME and GP17 on SH-SY5Y cell viability and apoptosis. A and B. The toxic effect of AGK-7 and 2-ME treatment on SH-SY5Y cells. The cell viability measured at 490 nM by using the MTT assay (n = 6). C. The TUNEL staining in OGD/R-induced SH-SY5Y cells, measured by a fluorescence microscope, scale bar=200 μm. The data presented as Mean values ± SEM. ^#^P < 0.01, ^##^P < 0.01 versus the control group.

**
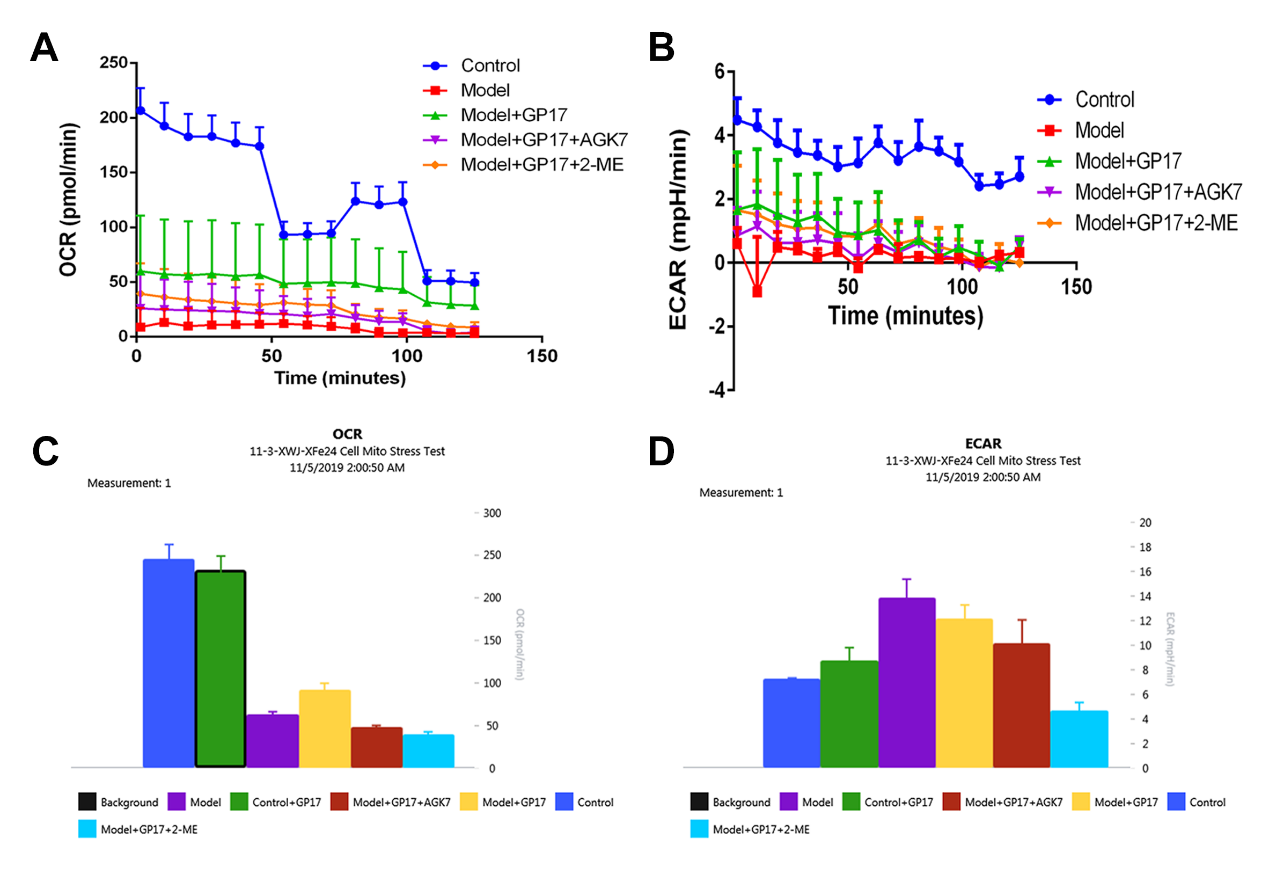
**

**Fig. S3** Effects of GP17 on the cell energy metabolism in OGD/R-induced SH-SY5Y cells, which partly reversed by the inhibitors AGK-7 and 2-ME. A. Effects of GP17 on the mitochondrial respiratory and glycolysis OCR levels in OGD/R-induced SH-SY5Y cells, detected by the Seahorse XFp Extracellular Flux Analyzer. B. Effects of GP17 on the mitochondrial respiratory and glycolysis OCR in OGD/R-induced SH-SY5Y cells, detected by the Seahorse XFP Extracellular Flux Analyzer. C and D. The statistics values of OCR and ECAR levels presented by the Seahorse XFP Extracellular Flux Analyzer. The data, presented as Mean values ± SEM (n=3-4).
